# Fermentation and kinetics characteristics of a bioflocculant from potato starch wastewater and its application

**DOI:** 10.1038/s41598-018-21796-x

**Published:** 2018-02-26

**Authors:** Junyuan Guo, Jianying Liu, Yijin Yang, Yuling Zhou, Shilin Jiang, Cheng Chen

**Affiliations:** College of Resources and Environment, Chengdu University of Information Technology, Chengdu, Sichuan 610225 China

## Abstract

Potato starch wastewater was used as fermentation medium for *Rhodococcus erythropolis* to produce bioflocculant. Kinetics of cell growth and bioflocculant production were firstly constructed. After fermentation for 60 h, 0.97 g of bioflocculant with polysaccharides nature was extracted from 1 L of fermentation liquor. Kinetics characteristics showed that cell growth and bioflocculant production could be simulated well with Logistic and Luedeking-Piret equations, respectively. When *R. erythropolis* was in logarithm growth phase, COD, ammonium, and TP of the potato starch wastewater medium were rapidly down to 1736, 188, and 146 mg/L, respectively, from 7836, 975, and 712 mg/L, while the medium’s exactly pH value was almost not changed. Furthermore, bioflocculant flocculation can be used as an effective pretreatment way for potato starch wastewater, and it was feasible in actual treatment projects in Ronghua Starch Co., Ltd., Sichuan Province.

## Introduction

Flocculation technique, an effective method in aggregating colloids, has been widely used in industrial settings^1^, for instance, a coagulation–flocculation (CF) process using iron-based coagulants resulted in COD, color, turbidity and humic acids removal of 56.4%, 63.4%, 89.8%, and 70.4% from landfill leachate^2^; after treated by the composite of bioflocculant and CaCl_2_, 91.8% of turbidity and 89.7% of COD were removed from domestic wastewater^3^. Although traditional flocculants were effective and economical in wastewater treatment, their degraded monomers always posed serious health and environmental concerns^4,5^. Compared with traditional flocculants, bioflocculant, secreted by microorganisms during their active secretion and cell lysis, has been attracted scientific attention due to its biodegradable and less harmful to human and ecosystems. Literatures reported that the bioflocculant was effective in the treatment of low temperature drinking water^6^, swine wastewater^7^, and so on, on laboratory scales.

Over time, different kinds of microorganisms and their bioflocculant have been reported, such as *Bacillus licheniformis*, *Lipomyces starkeyi*, and *Ochrobactium ciceri*^6,8,9^. However, there were no comprehensive studies on variation of the fermentation medium’s composition, which directly related to the characteristics of cell growth, metabolites generation, and fermentation kinetics^10^. It is precisely that the investigation of these elements would be beneficial for improving bioflocculant yield and flocculating activities in practical application^10^. Besides, high production cost was still the major limitation for bioflocculant in large scale production and commercial applications, and so far, with the aim of commercialization, a considerable effort has gone into reducing the production cost through using some wastes rich in organic matter, nitrogen, and phosphorus. For instance, Guo *et al*. produced bioflocculant by using swine wastes and rice stover^11^, Wang *et al*. produced bioflocculant by using dairy wastewater^12^, More *et al*. produced bioflocculant by using excess sludge^13^.

Potato starch wastewater, usually produced in the manufacturing process of potato starch and some related products, was one of the most serious and unwieldy pollution sources in food industry, in which the large amount of organic pollutants could be used to cultivate microorganisms to produce bioflocculant^14^. Utilization of potato starch wastewater to produce bioflocculant, can reduce the production cost, and further reduce the pollution caused by uncontrolled emissions of this type of wastewater.

This study effectively applied *R. erythropolis* to produce bioflocculant by using potato starch wastewater. Based on the optimization of the fermentation process, kinetics of cell growth and bioflocculant production were simulated. Meanwhile, concentrations of COD, ammonium, total phosphorus (TP), and pH value of the potato starch wastewater medium were examined. Furthermore, actual applications of the bioflocculant were discussed.

## Results

### Optimization of the fermentation process

**Figure 1:**
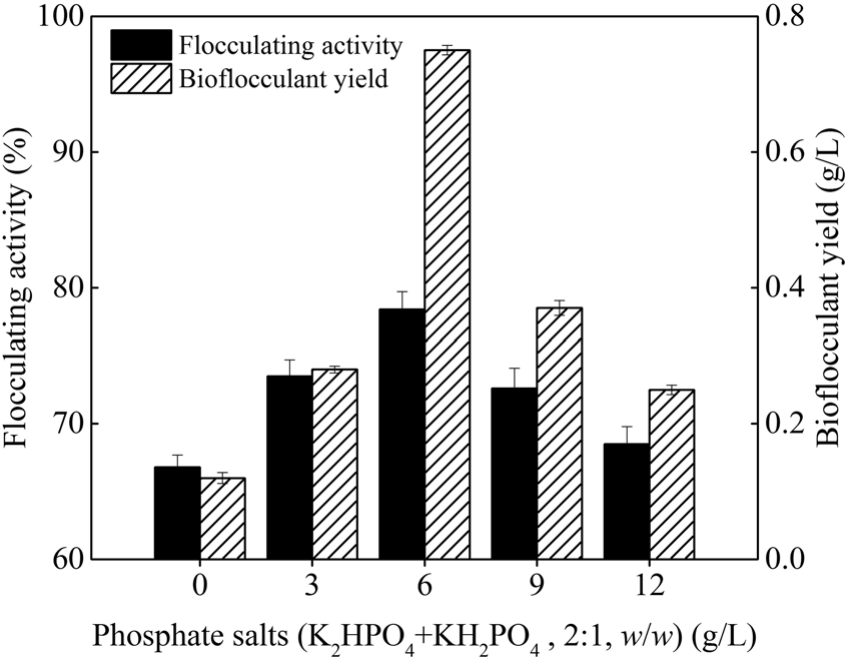
Effects of phosphate salts on bioflocculant yield and its flocculating activity (Major experimental conditions: *R. erythropolis* was incubated in the potato starch wastewater with 0.2 g/L MgSO_4_, 0.1 g/L NaCl, and different concentration of phosphate salts, with pH value of 6.8, at 150 r/min and 30  °C).

**Figure 2:**
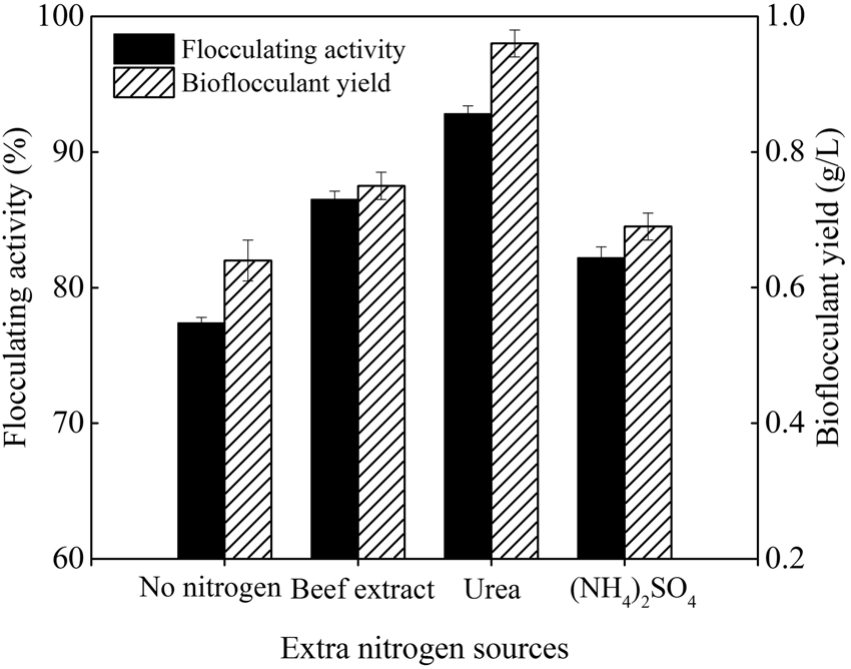
Effects of extra nitrogen sources on bioflocculant yield and its flocculating activity (Major experimental conditions: *R. erythropolis* was incubated in the potato starch wastewater with 4 g/L K_2_HPO_4_, 2 g/L KH_2_PO_4_, 0.2 g/L MgSO_4_, 0.1 g/L NaCl, and 2 g/L of different extra nitrogen, with pH value of 6.8, at 150 r/min and 30 °C).

**Figure 3:**
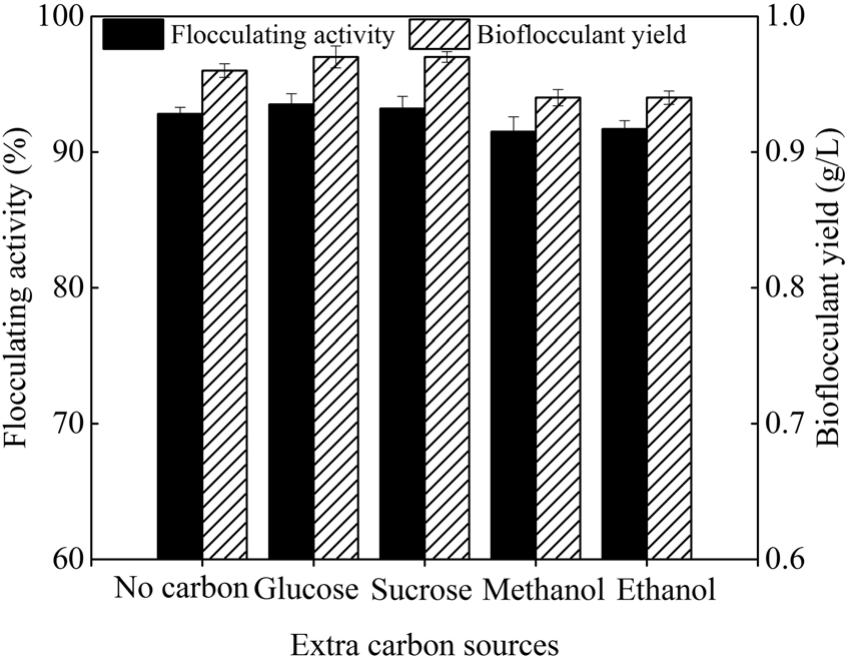
Effects of extra carbon sources on bioflocculant yield and its flocculating activity (Major experimental conditions: *R. erythropolis* was incubated in the potato starch wastewater with 4 g/L K_2_HPO_4_, 2 g/L KH_2_PO_4_, 0.2 g/L MgSO_4_, 0.1 g/L NaCl, 2 g/L urea, and 2 g/L of different extra carbon, with pH value of 6.8, at 150 r/min and 30 °C).

**Figure 4:**
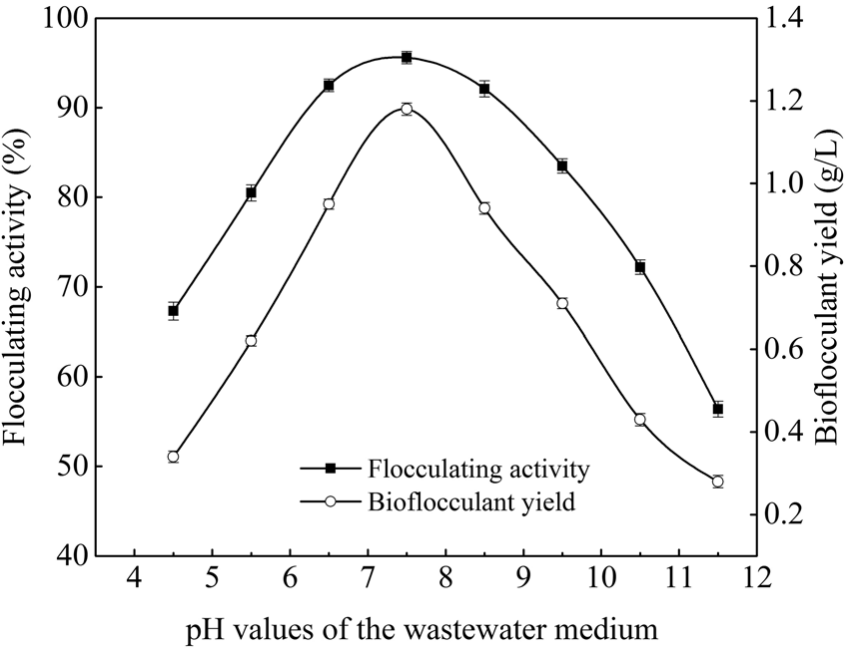
Effects of pH value of the wastewater medium on bioflocculant yield and its flocculating activity (Major experimental conditions: *R. erythropolis* was incubated in the potato starch wastewater medium of composition: 1 L potato starch wastewater, 4 g K_2_HPO_4_, 2 g KH_2_PO_4_, 0.2 g MgSO_4_, 0.1 g NaCl, and 2 g urea, with different pH value (4.5–11.5), at 150 r/min and 30 °C).

Reference to our previous study about the cultivation of *Paenibacillus polymyxa*^15^, effects of extra phosphate salts, nitrogen source, carbon source, and initial pH value of the potato starch wastewater medium on bioflocculant production were investigated in turn. From Figs 1–4, it is clearly showed that extra phosphate salts (at different dose) and all of the selected inorganic and organic nitrogen sources (including 2 g/L of (NH_4_)_2_SO_4_, urea, and yeast extract) were beneficial for bioflocculant yield and flocculating activity, while extra carbon sources (including 2 g/L of glucose and sucrose, and 2 mL/L of methanol and 95% ethanol) almost have no promotion, compared with the fermentation liquor from the potato starch wastewater medium (0.12 g/L of bioflocculant and 66.8% of flocculating activity can be obtained). Especially, the bioflocculant yield and flocculating activity improved to 0.75 g/L and 78.4% respectively when the total added phosphate salts were 6 g/L (4 g/L of K_2_HPO_4_ and 2 g/L KH_2_PO_4_), which were further improved to 0.96 g/L and 92.8% respectively when 2 g/L urea was extra added as the nitrogen source. The role of carbon during the bioflocculant production was inconsistent with conclusions reported by Xia *et al*. and Li *et al*., in which glucose was the most preferred carbon source for *Proteus mirabilis* and *Bacillus licheniformis*^6,16^. This may be due to the high-levels of organics in potato starch wastewater. When adjusted the initial pH value in range of 6.5–8.5, *R. erythropolis* can effectively produce bioflocculant, especially at pH point of 7.5, bioflocculant yield and flocculating activity were appeared as 1.18 g/L and 95.6%, respectively. It is noteworthy that pH range of 6.5–8.5 just covered the pH value of the potato starch wastewater (6.8), so, pH adjustment was not needed from a practical standpoint.

### Time course of cell growth and bioflocculant yield

**Figure 5:**
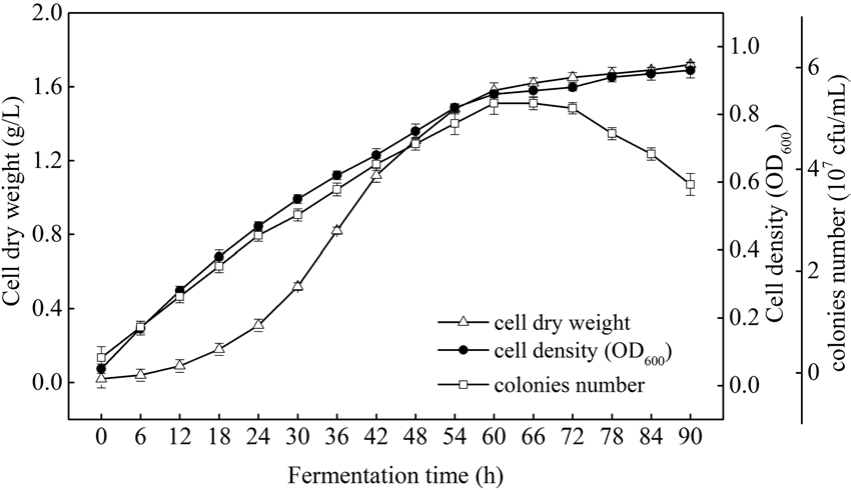
Growth curve of *Rhodococcus erythropolis* (Major experimental conditions: *R. erythropolis* was incubated in the potato starch wastewater medium of composition: 1 L potato starch wastewater, 4 g K_2_HPO_4_, 2 g KH_2_PO_4_, 0.2 g MgSO_4_, 0.1 g NaCl, and 2 g urea, with pH value of 6.8, at 150 r/min and 30 °C).

**Figure 6:**
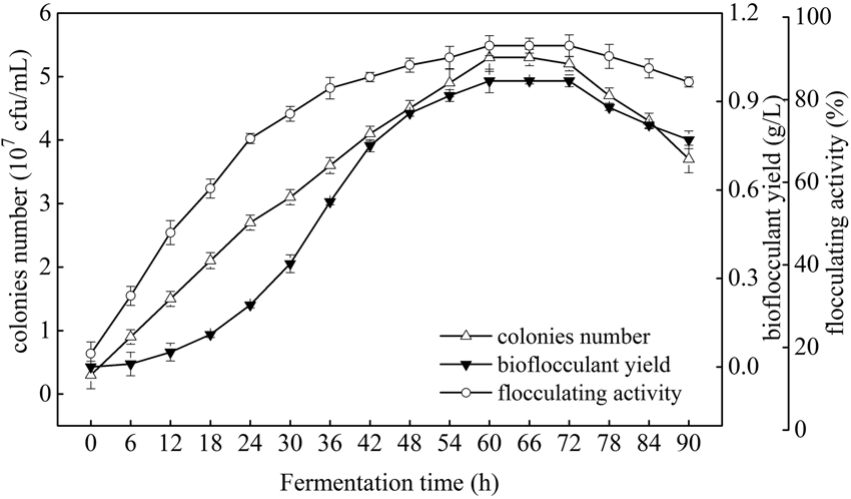
Relations between cell growth and bioflocculant production (Major experimental conditions: *R. erythropolis* was incubated in the potato starch wastewater medium of composition: 1 L potato starch wastewater, 4 g K_2_HPO_4_, 2 g KH_2_PO_4_, 0.2 g MgSO_4_, 0.1 g NaCl, and 2 g urea, with pH value of 6.8, at 150 r/min and 30 °C).

As seen from the cell growth curve in Fig. 5, after cultivation for 60 h, cell dry weight, cell density (OD_600_), and colonies number were increased rapidly to 1.58 g/L, 0.86 and 5.3 × 10^7^ cfu/mL, respectively. Cells entered stationary phase since 60 h, and on 78 h and onward, the cells were in death phase, cell dry weight and density were still increased, while colonies number was reduced. As known to all, cell dry weight contained both the weight of live and death bacteria, so it was increased. The increasing in cell density was due to the increased turbidity after bacteria died in the medium. Colonies number was expressed in the number of viable cells, which was reduced when the cells were entered into death phase, due to the rapid reduction of carbon source, ammonium, and phosphorus, and the cytotoxicity chemicals derived from the accumulation of metabolites in medium^17^. From Fig. 6, both of bioflocculant yield and its corresponding flocculating activity were increased along with the fermentation time and reached their peaks of 0.97 g/L and 93.1% at 60 h as well.

Above results reflected that cell growth as well as bioflocculant yield increased sharply in the period of 0–60 h. Both bioflocculant yield and colonies number were reached their peaks at 60 h and then stabilized after 60 h, indicated that bioflocculant production was almost simultaneously accompanied with cell growth. Afterwards, the bioflocculant yield was decreased monotonically to 0.77 g/L at 90 h, which may be due to cell autolysis and the decrease of enzymatic activity^9^. A similar bioflocculant production style was observed by *Aspergillus parasiticus*^18^.

### Analysis of the bioflocculant’s nature

Proportions of total sugar and protein contents were detected as 92.3% and 7.6% (w/w), respectively. Further chemical analysis revealed that the total sugar mainly including 49.7% of neutral sugar, 13.2% of uronic acid, 12.4% of amino sugar, and so on, different from our previous research in which bioflocculant was prepared by using swine wastes^11^. This was ascribed to the different nutrition components and biophysical environments, which may account for the discrepancy in bioflocculant components^19^. Gel permeation chromatography indicated that approximate molecular weight of the bioflocculant was 3.85 × 10^5^ Da.

Results of thermal stability test showed that the bioflocculant was thermostable and retained more than 89.7% of flocculating activity in a temperature range of 10–70 °C. Further, residual flocculating activities of 80.3% or more could still be achieved after heating for 30 min at 80, 100, and 120 °C, respectively. These thermo-stable characteristics suggested that active ingredients in the bioflocculant were mainly polysaccharide. Literatures reported that in temperature range of 30–120 °C, polysaccharide chains can remain extended and exposed its flocculating sites, and ensure the flocculating activity, for example, flocculating activity of the bioflocculant produced by *Bacillus subtilis* remained its 89% at 100 °C^20^.

Infrared spectrum in Fig. S1 displayed a broad stretching peak at 3430 cm^−1^, which was assigned to –OH and –NH_2_^21^, in accordance with that of NOC-1 (mainly glycoprotein)^18^. The peak around 1632 cm^−1^ may be assigned to the –CO stretching in –CONH_2_ group, and the peaks around 1080 cm^−1^ were characteristic of C–O groups. The peak at 1405 cm^−1^ is probably an indication of –COO^−^ symmetric stretching vibration. Typical functional groups of polysaccharide, namely, hydroxyl (–OH), amino (–NH_2_), carboxyl (C–O) groups, and acylamino (–CONH_2_) groups were all observed in the FTIR spectrum, revealed that the bioflocculant mainly contains polysaccharide. The FTIR spectrum of this bioflocculant was similar to that of Sun *et al*.’s research, in which functional groups of a polysaccharide flocculant made from sludge cells directly were investigated^21^. But the bioflocculant was somehow different from the *R. erythropolis* sludge bioflocculant (RSF), which was prepared by using sludge and livestock wastewater^19^, even though the FTIR spectrum of the RSF showed the typical functional groups of polysaccharide, the former exhibited absorption at 582 cm^−1^, corresponding to the existence of protein.

### Variation of the medium’s composition

**Figure 7:**
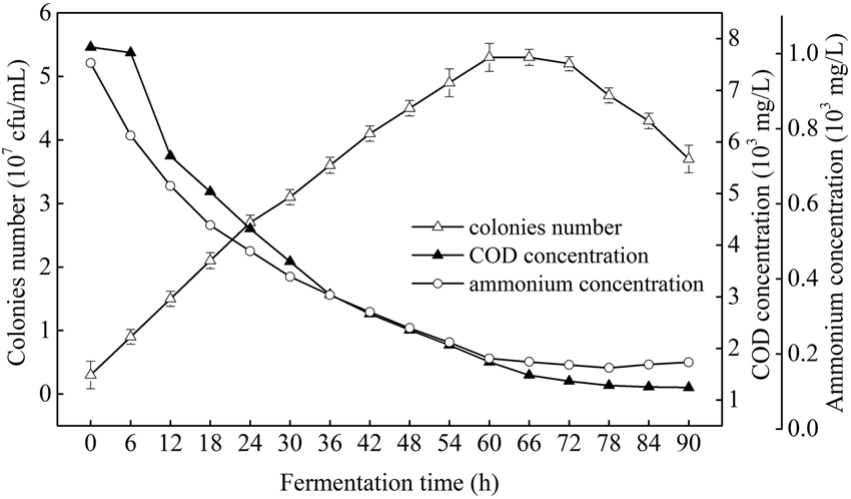
Changes of COD and ammonium with cell growth during the fermentation process (Major experimental conditions: *R. erythropolis* was incubated in the potato starch wastewater medium of composition: 1 L potato starch wastewater, 4 g K_2_HPO_4_, 2 g KH_2_PO_4_, 0.2 g MgSO_4_, 0.1 g NaCl, and 2 g urea, with pH value of 6.8, at 150 r/min and 30 °C).

**Figure 8:**
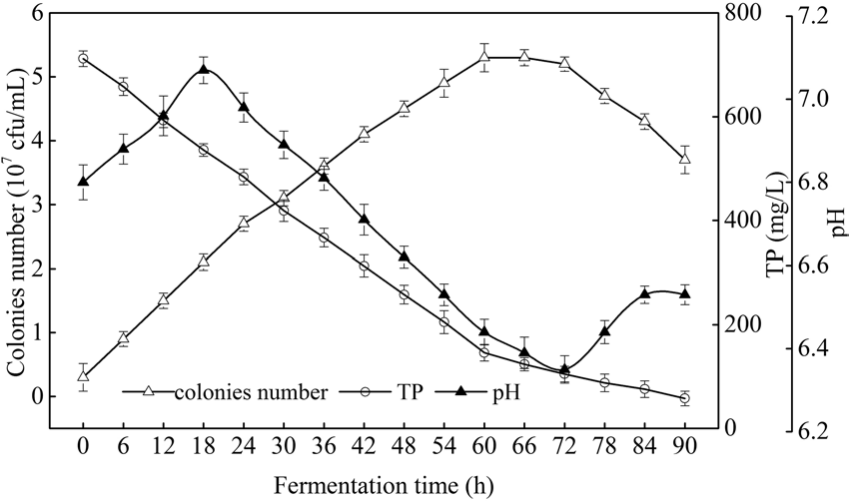
Changes of TP and pH value with cell growth during the fermentation process (Major experimental conditions: *R. erythropolis* was incubated in the potato starch wastewater medium of composition: 1 L potato starch wastewater, 4 g K_2_HPO_4_, 2 g KH_2_PO_4_, 0.2 g MgSO_4_, 0.1 g NaCl, and 2 g urea, with pH value of 6.8, at 150 r/min and 30 °C)

During fermentation process, COD and ammonium contents were rapidly down to 1736 and 188 mg/L from 7836 and 975 mg/L, respectively when *R. erythropolis* was in logarithm growth phase, while in stationary phase (60–78 h), the down-ward trends of COD and ammonium were became slowly and maintained at a low level at last (Fig. 7). Further, ammonium was recovered slowly after *R. erythropolis* entered into death phase. From Fig. 8, it is clearly showed that TP concentration was decreased rapidly to 146 mg/L when *R. erythropolis* was in logarithm growth phase, and the downward trend was just slow after entered into the stationary and death phases. In addition, exactly pH value of the potato starch wastewater medium was increased first and then decreased but the trend was not significant (maintained in the range of 6.4–7.0), due to the buffer effect of phosphorus^15^.

### Cell growth kinetics

**Figure 9:**
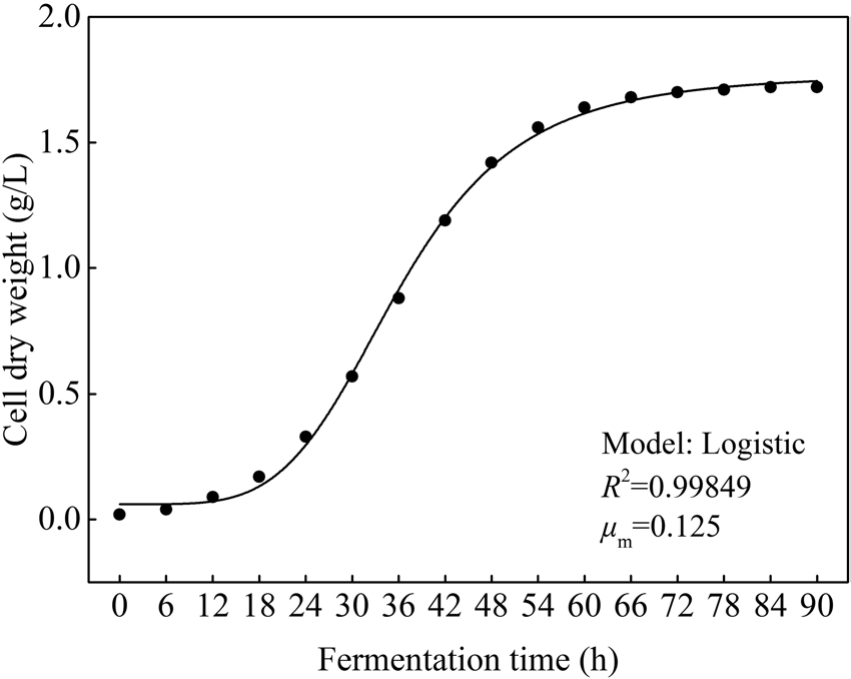
Fitting curve of cell growth kinetics which described the cell dry weights (Major experimental conditions: *R. erythropolis* was incubated in the potato starch wastewater medium of composition: 1 L potato starch wastewater, 4 g K_2_HPO_4_, 2 g KH_2_PO_4_, 0.2 g MgSO_4_, 0.1 g NaCl, and 2 g urea, with pH value of 6.8, at 150 r/min and 30 °C).

During fermentation process, the initial and the largest cell dry weights (*X*_0_, *X*_m_) were detected as 0.02 and 1.72 g/L, respectively. Cell growth kinetics which described the cell dry weights was obtained from the non-linear fit (Fig. 9) of Eq. (5) and given as follows:

1 $$X({\rm{t}})=\frac{1.72}{1+{{\rm{e}}}^{4.45-0.125t}}$$

**Figure 10:**
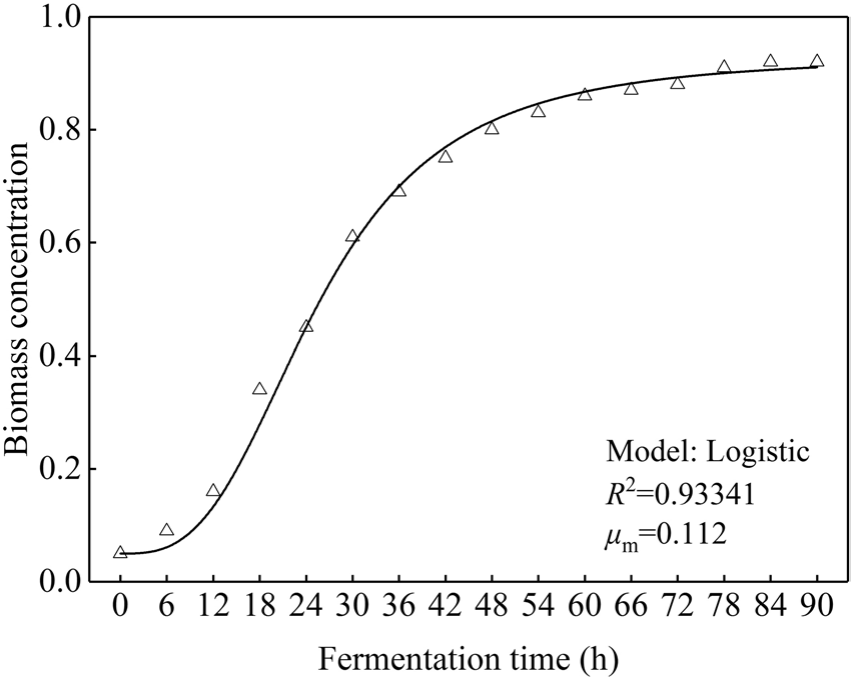
Fitting curve of cell growth kinetics which described the biomass concentrations (Major experimental conditions: *R. erythropolis* was incubated in the potato starch wastewater medium of composition: 1 L potato starch wastewater, 4 g K_2_HPO_4_, 2 g KH_2_PO_4_, 0.2 g MgSO_4_, 0.1 g NaCl, and 2 g urea, with pH value of 6.8, at 150 r/min and 30 °C).

During fermentation process, the initial and the largest biomass concentrations (*X*_0_, *X*_m_) were detected as 0.05 and 0.92, respectively. Cell growth kinetics which described the biomass concentrations was obtained from the non-linear fit (Fig. 10) of Eq. (5) and given as follows:

2 $$X({\rm{t}})=\frac{0.92}{1+{{\rm{e}}}^{2.91-0.112t}}$$

From Table 1, the average relative error between the cell dry weights predicted by the cell growth kinetics model (predicted value) and measured from experiments (actual value) was appeared as 3.98% <10%, similarly, the average relative error between the biomass concentrations predicted by the cell growth kinetics model (predicted value) and measured from experiments (actual value) was appeared as 3.19% <10%. In addition, *R*^2^ of Eqs (1) and (2) were 0.99849 and 0.93341, both higher than 0.90 (Figs 9 and 10). The above information indicated that cell growth kinetics model both described the cell dry weights and biomass concentrations constructed using Logistic equation can well explain the growth characteristics of *R. erythropolis*.

**Table 1:** Predicted and experimental values of the dry weight and biomass concentration.

Fermentation time (h)	Cell dry weight (g/L)			Biomass concentration		
	Experimental value	Predicted value	Relative error (%)	Experimental value	Predicted value	Relative error (%)
0	0.02	0.02	0	0.05	0.05	0
6	0.04	0.04	0	0.09	0.08	0
12	0.09	0.09	0	0.16	0.16	0
18	0.18	0.17	5.56	0.34	0.33	2.94
24	0.31	0.33	6.45	0.45	0.41	8.51
30	0.52	0.57	9.62	0.61	0.56	8.20
36	0.82	0.88	7.32	0.69	0.69	0
42	1.12	1.19	6.25	0.75	0.79	5.33
48	1.31	1.42	8.40	0.80	0.85	6.25
54	1.48	1.56	5.41	0.83	0.88	6.02
60	1.58	1.64	3.80	0.86	0.90	4.65
66	1.62	1.68	3.70	0.87	0.91	4.60
72	1.65	1.70	3.00	0.88	0.91	3.41
78	1.67	1.71	2.40	0.91	0.92	1.10
84	1.69	1.72	1.78	0.92	0.92	0
90	1.72	1.72	0	0.92	0.92	0

### Bioflocculant synthesis kinetics

In this study, the bioflocculant production was almost simultaneously accompanied with cell growth, thus, the integration of Eq. (6) can be lead to:

3 $$P({\rm{t}})+{m}_{{\rm{1}}}[\frac{{X}_{{\rm{m}}}}{{\rm{1}}+{{\rm{e}}}^{[\mathrm{ln}({X}_{{\rm{m}}}/{X}_{{\rm{0}}})-{\mu }_{{\rm{m}}}t]}}-{X}_{{\rm{0}}}]$$

**Figure 11:**
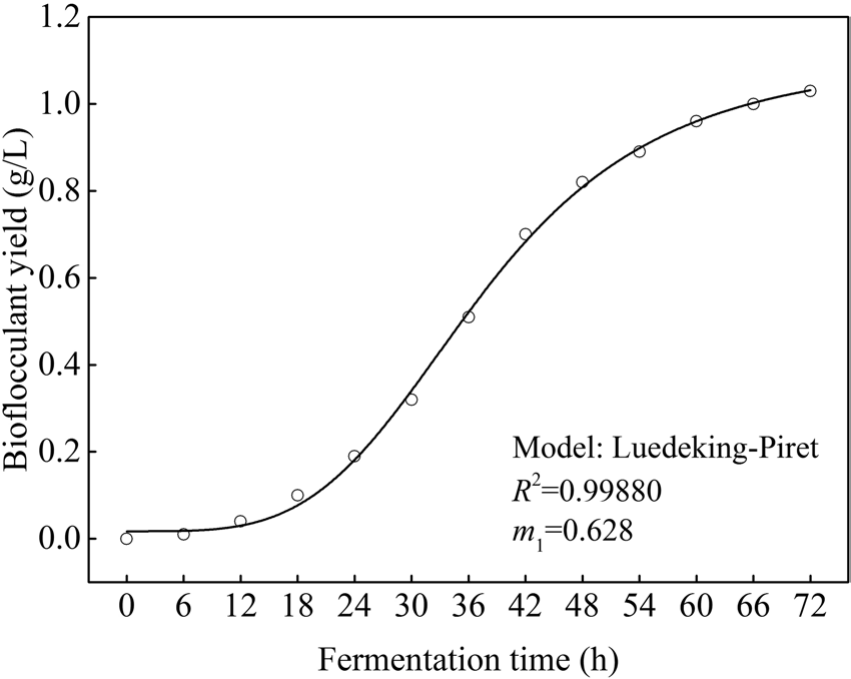
Fitting curve of bioflocculant synthesis kinetics (Major experimental conditions: *R. erythropolis* was incubated in the potato starch wastewater medium of composition: 1 L potato starch wastewater, 4 g K_2_HPO_4_, 2 g KH_2_PO_4_, 0.2 g MgSO_4_, 0.1 g NaCl, and 2 g urea, with pH value of 6.8, at 150 r/min and 30 °C).

Bioflocculant synthesis kinetics was obtained from the non-linear fit (Fig. 11) of Eq. (3) and given as follows:

4 $$P({\rm{t}})=0.628[\frac{1.72}{1+{{\rm{e}}}^{4.45-0.125t}}-0.02]$$

From Table 2, the average relative error between the bioflocculant yields predicted by the kinetics model (predicted value) and measured from experiments (actual value) was appeared as 4.69% <10% when the strain was in the logarithm growth and stationary phases, and the *R*^2^ was appeared as 0.99880 >0.90 (Fig. 11), indicated that the bioflocculant synthesis kinetics model constructed using Luedeking-Piret equation can well explain the bioflocculant production process by *R. erythropolis*.

**Table 2:** Predicted and experimental values of the bioflocculant yields.

Fermentation time (h)	Experimental value (g/L)	Predicted value (g/L)	Relative error (%)
0	0	0	0
6	0.01	0.01	0
12	0.04	0.04	0
18	0.11	0.10	9.09
24	0.21	0.19	9.52
30	0.35	0.32	8.57
36	0.56	0.51	8.93
42	0.75	0.70	6.67
48	0.86	0.82	4.65
54	0.92	0.89	3.26
60	0.97	0.96	1.03
66	0.97	1.00	3.09
72	0.97	1.03	6.17

### Preliminary application in pretreatment of potato starch wastewater

**Figure 12:**
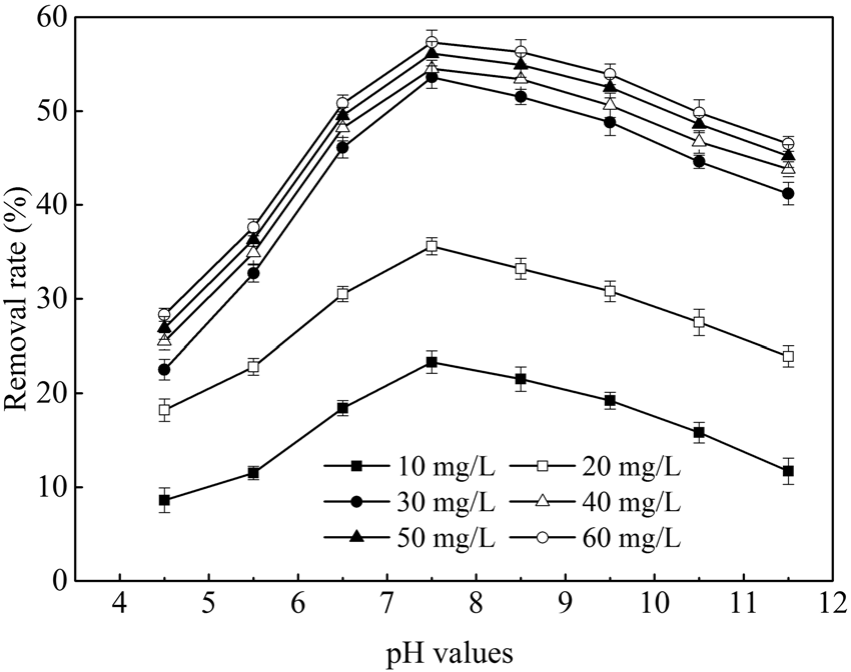
Effects of bioflocculant doses on COD removal under different pH values of the potato starch wastewater (Major experimental conditions: bioflocculant doses 10–60 mg/L, solution pH values 4.5–11.5).

Figure 12 depicted COD removal at different bioflocculant doses and solution pH values. Under different solution pH values (4.5–11.5), COD removal rates achieved their maximum value at bioflocculant dose of 30 mg/L. In addition, the bioflocculant showed good performances in weak acid, weak alkaline and neutral pH conditions (6.5–9.5) for the removal of COD from potato starch wastewater. Especially, at solution pH point of 7.5, after treated by 30 mg/L of the bioflocculant, COD removal rate can reached 57.3%, which was decreased beyond this pH value. Similar conclusions for potato starch wastewater treatment by *Paenibacillus polymyxa* and its bioflocculant have been reported^15^.

Based on the good performance in pretreatment of potato starch wastewater in laboratory, real application scenario of the bioflocculant was investigated. Currently, in Ronghua Starch Co., Ltd., Sichuan Province, a sequencing batch reactor (SBR) was constructed to treat potato starch wastewater, due to the better anti-shock loading capability and system stability. Under the condition of influent average COD of 7836 mg/L, the temperature of 25 °C, continual aerations of 2.5 h and sludge density of 2000–3000 mg/L, the reactor had a good degeneration ability of COD and ammonium in potato starch wastewater, and the corresponding removal rates could reach 65% or more (without addition of bioflocculant) (Table 3). After treated under the same condition and at the same procedure with addition of 30 mg/L bioflocculant during a long period of 60 d, the average removal rates increased up to 97.3% for COD and 94.6% for ammonium.

**Table 3:** Qualities of potato starch wastewater before and after treated by SBR (with or without bioflocculant addition) (under the condition of influent average COD of 7836 mg/L, temperature of 25 °C, continual aerations of 2.5 h and sludge density of 2000–3000 mg/L)

	Potato starch wastewater	After treated by SBR technology without bioflocculant	Removal rate (%)	After treated by SBR technology with 30 mg/L bioflocculant	Removal rate (%)
COD (mg/L)	7836	2492	68.2	211.6	97.3
BOD_5_ (mg/L)	2154	747.4	65.3	176.6	91.8
**Ammonium**					
(mg/L)	25.6	8.7	66.1	1.4	94.6
**TP**					
(mg/L)	18.3	7.2	60.8	0.27	98.5
Turbidity (NTU)	685	187.7	72.6	38.4	93.4
Chroma	45	13.3	70.5	2.7	94.0
pH value	6.8	6.5	—	6.6	—

## Discussion

A bioflocculant was harvested by culturing *R. erythropolis* in potato starch wastewater medium, the optimum medium composition and the main backbone of this bioflocculant were similar with previous reports from this lab, in which *Paenibacillus polymyxa* was selected as the bioflocculant-producing strain^15^. The bioflocculant yield of 0.97 g/L was higher than that reported in similar previous researches^14,22^. How about this yield compared to that using normal medium? Under the same fermentation process in standard fermentation medium as peptone 10 g, beef extracts 3 g, K_2_HPO_4_ 4 g, KH_2_PO_4_ 2 g, MgSO_4_ 2 g, and NaCl 5 g dissolved in 1 L distilled water with the pH value of 6.8, 1.12 g of bioflocculant can be extracted from 1 L of fermentation liquor after fermentation for 60 h, an increasing of about 15.5%, compared with the 0.97 g/L. This result suggested that the potato starch wastewater could be an effective alternative medium to produce bioflocculant.

Growth curve of *R. erythropolis* showed that the bioflocculant formation was almost simultaneously accompanied with cell growth, similar with the fermentation of *Aspergillus parasiticus*^18^. Cell growth and bioflocculant production could be simulated with both Logistic and Luedeking-Piret equations pretty well. COD, ammonium, and TP of the potato starch wastewater medium were rapidly down to 1736, 188, and 146 mg/L, respectively, while the exactly pH value was almost no change. The decline of COD, ammonium, and TP may be due to the fast utilization rate of organic matter and nitrogen by *R. erythropolis* in logarithm growth phase. Ammonium recovered slowly after the strain entered into death phase, in this phase, cell death and autolysis resulting in the releasing of intracellular protein into the wastewater medium^17^.

Further, from a practical standpoint, at natural pH value of the potato starch wastewater of 6.8, after treated by 30 mg/L bioflocculant, the residual COD, BOD_5_, ammonium, TP, turbidity, and chroma of the potato wastewater were detected as 3652.6, 883.4, 10.2, 8.5 mg/L, 247 NTU, and 15.7 times, which were decreased a lot compared with the initial concentrations (7836, 2154, 25.6, 18.3 mg/L, 685 NTU, 45 times), indicated that the bioflocculant flocculation can be used as an effective pretreatment way for the potato starch wastewater. The improvement of removal rate of COD from 68.2% to 97.3% and ammonium from 66.1% to 94.6% when the 30 mg/L of bioflocculant was applied to combine with the sequencing batch reactor (SBR), indicated that the bioflocculant was feasible in actual projects of potato starch wastewater treatment in Ronghua Starch Co., Ltd., Sichuan Province. Some other bacteria were also effective in pollutants removal from wastewaters, for example, *Coelastrella* sp. QY01 can remove 90% of ammonium and 90% of TP from aerobically treated swine wastewater after cultivated for 10 days^23^; *Rhodobacter blasticus and Rhodobacter capsulatus* can remove 83.3% of COD from aerobically treated swine wastewater after cultivated for 72 h^24^.

## Materials and Methods

### Potato starch wastewater

Potato starch wastewater used in this study was taken from Ronghua Starch Co., Ltd., Sichuan Province, and its water quality was shown in Table 4.

**Table 4:** Qualities of potato starch wastewater and potato starch wastewater medium.

	Potato starch wastewater	Potato starch wastewater medium
COD (mg/L)	7836	7836
BOD_5_ (mg/L)	2154	2154
Ammonium (mg/L)	25.6	975
Total phosphorous (mg/L)	18.3	732
Turbidity (NTU)	685	—
Chroma	45	38
pH value	6.8	6.8

### Bioflocculant production

*R. erythropolis*, deposited in China Center for Type Culture Collection (No. 10543), was specified to produce bioflocculant. Before bioflocculant production, *R. erythropolis* was first inoculated in 150 mL seed medium consisted of peptone 10 g, beef extracts 3 g, and NaCl 5 g dissolved in 1 L distilled water, and incubated on a reciprocal shaker (SHA-A, Shanghai Lianhua Company, China) at 150 r/min and 30 °C for 24 h. After cultivation, 2.0% v/v of the above inoculums was used to inoculate the potato starch wastewater medium of composition: 1 L potato starch wastewater, 4 g K_2_HPO_4_, 2 g KH_2_PO_4_, 0.2 g MgSO_4_, 0.1 g NaCl, and 2 g urea, and incubated in the same procedure to produce bioflocculant. Main characteristics of the potato starch wastewater medium was also shown in Table 4.

After fermentation for 60 h, the fermentation liquor was obtained, from the whole of which the bioflocculant was extracted by using the methods proposed by Guo *et al*.^11^, with the following modifications: the fermentation liquor with flocculating components was centrifuged at 3000 r/min for 30 min to obtain supernatant containing slime bioflocculant and pellet containing capsular bioflocculant. To determine the slime bioflocculant, the supernatant was precipitated with 2 volumes of chilled absolute acetone (containing 0.07% *β*-mercaptoethanol) by incubating the mixture at −20 °C for 4 h, then, the resulting precipitate was collected by centrifugation at 3000 r/min for 30 min and the slime bioflocculant was obtained. To determine the capsular bioflocculant, the pellet was re-suspended in phosphate solution, and heated at 40 °C for 15 min followed by centrifugation at 3000 r/min for 30 min, then, the supernatant was treated in the same the same procedure as the determination of slime bioflocculant to obtain the capsular bioflocculant. Sum of the slime and the capsular was denoted as the bioflocculant produced by *Rhodococcus erythropolis* in this study.

### Physical and chemical analysis of the bioflocculant

Total sugar content of the purified bioflocculant was determined by the phenol-sulfuric acid method^25^. Protein content was measured by the Bradford method^26^. Molar mass was determined by gel permeation chromatography (GPC) using a Hitachi L-6200 system controller. Functional groups were determined by using a Fourier transform infrared spectrophotometer (EQUINOX 55, Bruker Company, Germany). Thermal stability of the bioflocculant was determined as follows: the bioflocculant was dissolved in a suitable volume of deionized water to achieve an initial flocculating activity of over 90% and divided into 12 aliquots, which were placed at 10, 20, 30, 40, 50, 60, 70, 80, 90, 100, 110, and 120 °C for 30 min, respectively. Subsequently, their flocculating activities were measured. The thermal stability was judged according to the changeable of the flocculating activities.

### Assay of flocculating activity

Flocculating activity was measured in jar tests referenced to the method reported in our previous publication^15^, in which 4 g/L of kaolin clay suspension was chosen as the suspended solid, whose optical density (OD) were measured with a spectrophotometer (Unic–7230, Shanghai Lianhua Company, China) at 550 nm before and after treated by the bioflocculant. The control experiment was conducted in the same manner without adding bioflocculant.

### Determination of biomass concentration, colonies number, and cell dry weight

Biomass concentration was measured by turbidimetry method using a spectrophotometer (Unic–7230, Shanghai Lianhua Company, China) at 600 nm, numbers of colonies were counted using the plate count method^17^. Cell dry weight was determined as follows: the fermentation liquor at different fermentation time was centrifuged at 3000 r/min for 10 min, and the resulting precipitate (strain cell) was collected and then dried in an oven at 105 °C until a constant weight, which was denoted as cell dry weight.

### Determination of COD, ammonium, and TP

COD, ammonium, and TP of the potato starch wastewater medium were determined according to the EPA Standard Methods^27^.

### Fermentation kinetics

Generally, Logistic equation could nicely describe the dynamic process of cell growth, and was normally given as follows:

5 $$X({\rm{t}})={X}_{{\rm{m}}}/[{\rm{1}}+{{\rm{e}}}^{{\rm{In}}{}^{({X}_{{\rm{m}}}/{X}_{{\rm{0}}})-{\mu }_{{\rm{m}}}t}}]$$

where *X*_0_ and *X*_m_ were the initial and the maximum cell dry weight (or biomass concentration), respectively; *X*(t) was the cell dry weight (or biomass concentration) at fermentation time *t* (g/L); *μ*_m_ was a constant that characterized the maximum specific growth rate (h^−1^); *t* was the fermentation time (h).

Luedeking-Piret equation was always applied to simulate the process of bioflocculant production, and was normally given as follows:

6 $$\frac{{\rm{d}}P}{{\rm{d}}t}={m}_{1}X+{m}_{2}\frac{{\rm{d}}X}{{\rm{d}}t}$$

Where *P* was the bioflocculant yield (g/L); *m*_1_ and *m*_2_ were the equation parameters, they were constants when the fermentation conditions were determined.

### Actual application

Potato starch wastewater from Ronghua Starch Co., Ltd., Sichuan Province was selected to identify the potential of the bioflocculant in actual application. Bioflocculant dose and pH values of the potato starch wastewater were used to optimize the flocculating conditions. A sample of 1.0 L wastewater was poured in a beaker and the pH value was adjusted using 1.0 mol/L NaOH or HCl if necessary. The bioflocculant was then added, and the mixture was stirred at the design agitation speed for 10 min, and then allowed to stand 30 min. Referenced to the treatment process reported in our previous publication^15^, the residual COD, BOD_5_, ammonium, TP, turbidity, and chroma of the potato wastewater were determined after the pretreatment.

All the measurements through this study were carried out in triplicates and the average values were presented (with standard error less than 5% of the mean).

## Electronic supplementary material

Supplementary data
