# Supplementary material for: Fermentation and kinetics characteristics of a bioflocculant from potato starch wastewater and its application
Source: Sci Rep. 2018 Feb 26;8:3631. doi: 10.1038/s41598-018-21796-x (PMC5827517; doi:10.1038/s41598-018-21796-x)
Supplement: Supplementary file 1 — Supplementary data [file 41598_2018_21796_MOESM1_ESM.doc]

**Fermentation and kinetics characteristics of a bioflocculant from potato starch wastewater and its application**

**Junyuan Guo*, Jianying Liu, Yijin Yang, Yuling Zhou, Shilin Jiang, Cheng Chen**

College of Resources and Environment, Chengdu University of Information Technology, Chengdu, Sichuan 610225, China

*Correspondence and requests for materials should be addressed to J.G. (email: [gjy@cuit.edu.cn](mailto:yangc@hnu.edu.cn))


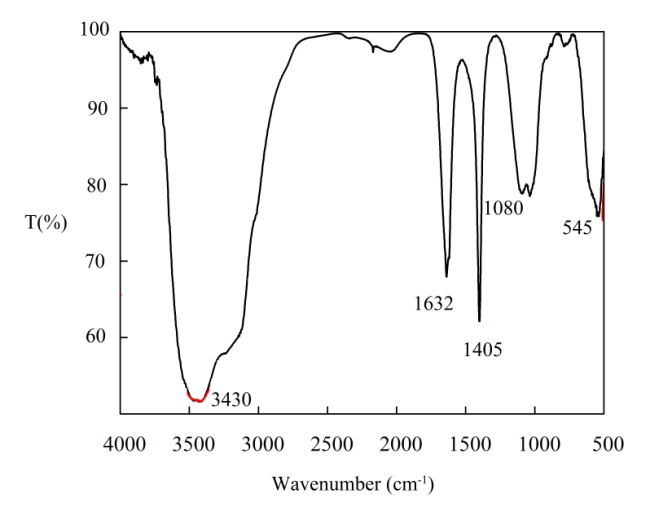


**Fig.S1** Fourier transform infrared (FTIR) spectrum of the bioflocculant
